# Microstructure of Nonjuxtapapillary Microvasculature Dropout in Healthy Myopic Eyes

**DOI:** 10.1167/iovs.61.2.36

**Published:** 2020-02-21

**Authors:** Gyu-Nam Kim, Eun Ji Lee, Tae-Woo Kim

**Affiliations:** 1 Department of Ophthalmology, Institute of Health Sciences, Gyeongsang National University School of Medicine, Gyeongsang National University Hospital, Jinju, South Korea; 2 Department of Ophthalmology, Seoul National University College of Medicine, Seoul National University Bundang Hospital, Seongnam, South Korea

**Keywords:** myopia, optical coherence tomography angiography, peripapillary microvasculature, primary open-angle glaucoma

## Abstract

**Purpose:**

The purpose of this study was to characterize the microstructure of the nonjuxtapapillary microvasculature dropout (MvD) in healthy myopic eyes.

**Methods:**

This cross-sectional study included 50 eyes (25 eyes with a nonjuxtapapillary MvD and 25 age-matched eyes without any MvD) from a cohort of 126 nonglaucomatous healthy myopic eyes having parapapillary atrophy (PPA) γ-zone. The parapapillary deep-layer microvasculature was evaluated in en-face images obtained using swept-source optical coherence tomography (OCT) angiography (OCTA). A nonjuxtapapillary MvD was defined as an area with focal absence of vascular signals in the distal portion of PPA confined to the nonjuxtapapillary area. Enhanced depth-imaging OCT scanning was performed to assess the parapapillary microstructure.

**Results:**

Nonjuxtapapillary MvD was found in 25 eyes (19.8%). The parapapillary microstructure at the nonjuxtapapillary MvD in 18 eyes was characterized by the misalignment of Bruch's membrane (BM)–retinal pigment epithelium (RPE) complex, which was identified by the absence of BM-RPE complex and the presence of the inner retina and sclera. In seven eyes with a nonjuxtapapillary MvD but without such misaligned BM-RPE complex, RPE atrophy was observed at the location of the nonjuxtapapillary MvD. Eyes with a nonjuxtapapillary MvD had a longer axial length (AXL; *P* = 0.013) and a wider γ-zone (*P* < 0.001) than age-matched control eyes without any MvD.

**Conclusions:**

The microstructure at the nonjuxtapapillary MvD in healthy myopic eyes was characterized in approximately 70% of eyes by temporally misaligned BM-RPE complex. Although the clinical importance of the nonjuxtapapillary MvD remains to be determined, it should be differentiated from the parapapillary choroidal MvD observed in glaucoma.

The juxtapapillary choroidal vasculature is closely associated with the perfusion of the optic nerve head (ONH) tissues. It has been shown that decreased perfusion in the juxtapapillary choroid is closely associated with glaucoma.^1^^,^^2^ Recent studies using optical coherence tomography (OCT) angiography (OCTA) demonstrated the presence of localized microvasculature dropout (MvD) in the juxtapapillary choroid in glaucomatous eyes,^3^^,^^4^ which was highly consistent with the location of the glaucomatous damage.^3^^,^^5^ The MvD identified in OCTA corresponded exactly to the perfusion defect shown by indocyanine-green angiography, suggesting that it represents a true perfusion defect in the juxtapapillary choroid.^5^

MvD has been exclusively found within the area of parapapillary atrophy (PPA) in glaucomatous eyes.^3^^,^^4^ PPA is divided into two distinctive structures based on the presence and absence of Bruch's membrane (BM) within the PPA area, which is referred to as the β- and γ-zones, respectively.^6^^,^^7^ The PPA γ-zone is characterized by an oblique scleral flange (SF) and is associated with myopia, and so it has been suggested that this zone develops via exposure of the SF as the myopic globe expands.^8^ Studies have found that the prevalence of MvD is higher in eyes having a γ-zone than in those without a γ-zone in the PPA area.^9^^,^^10^ However, the MvD in the γ-zone could not be considered a dropout of choroidal microvessels, because the choroid is not usually present in that zone. Rather, it was suggested that the MvD in the γ-zone develops by stretching of the microvessels in the SF during axial elongation.^9^^,^^10^ Such an MvD has been characterized by it occurring in the juxtapapillary area adjoining the optic disc margin, and by its high frequency in glaucomatous eyes while rarely being found in healthy myopic eyes.^9^^–^^12^

We recently observed a nonjuxtapapillary MvD-like region in the distal portion of the PPA γ-zone in myopic eyes. This region did not adjoin the optic disc margin and could also be found in healthy eyes. The purpose of the present study was to characterize the parapapillary microstructure of the juxtapapillary MvD observed in the distal portion of the PPA γ-zone in nonglaucomatous myopic eyes, and to determine the clinical characteristics associated with this type of MvD.

## Methods

This study investigated subjects without glaucoma who were enrolled in the Investigating Glaucoma Progression Study, which is an ongoing prospective study of patients with glaucoma being performed at the Glaucoma Clinic of Seoul National University Bundang Hospital. Written informed consent to participate was obtained from all of the included subjects. The study protocol was approved by the Institutional Review Board of Seoul National University Bundang Hospital and followed the tenets of the Declaration of Helsinki.

### Study Subjects

All participants underwent comprehensive ophthalmic examinations that included the best-corrected visual acuity, Goldmann Applanation Tonometry, a refraction test, slit-lamp biomicroscopy, gonioscopy, color disc photography, red-free fundus photography (EOS D60 digital camera; Canon, Utsunomiya, Japan), corneal curvature measurement (KR-1800, Topcon), central corneal thickness measurement (Orbscan II; Bausch & Lomb Surgical, Rochester, NY, USA), axial length (AXL) measurement (IOLMaster version 5; Carl Zeiss Meditec, Dublin, CA), spectral-domain (SD) OCT scanning of the circumpapillary retinal nerve fiber layer (RNFL) (Spectralis; Heidelberg Engineering, Heidelberg, Germany), standard automated perimetry (Humphrey Field Analyzer II 750 and 24-2 Swedish interactive threshold algorithm, Carl Zeiss Meditec), and OCTA (DRI-OCT Triton; Topcon, Tokyo, Japan).

To be included in the study, subjects were required to have AXL >24 mm and a parapapillary γ-zone. The γ-zone was defined as an area with the exposed SF devoid of a BM–retinal pigment epithelium (RPE) complex and choroidal tissue.^7^^,^^8^ The β-zone was defined as the area between the end of BM and the beginning of the RPE.^6^^,^^7^ Eyes also had to have an intraocular pressure (IOP) of ≤21 mm Hg with no history of elevated IOP, no glaucomatous disc appearance, a circumpapillary RNFL thickness within the normal range (as measured by SD-OCT), and a normal visual field (VF). The absence of a glaucomatous disc was defined as the neuroretinal rim appearing intact and without disc hemorrhages, notches, or localized pallor. The normal range for the circumpapillary RNFL thickness was defined as within the 95th percentile of the normative database, whereas the VF was considered normal when there was no glaucomatous VF defect or neurological defects.^13^^,^^14^

The exclusion criteria were a best-corrected visual acuity of <20/40, a spherical equivalent of <–12.0 diopters (D), a cylinder correction of <–3.0 D or >+3.0 D, a history of intraocular surgery (with the exception of uneventful cataract surgery), or the presence of retinal or neurological diseases.

### OCTA and Determination of the Presence of a Nonjuxtapapillary MvD

The optic nerve and parapapillary area were imaged using a commercially available swept-source OCTA system (DRI OCT Triton; Topcon) operating at a central wavelength of 1050 nm, an acquisition speed of 100,000 A-scans per second, and axial and transverse resolutions of 7 and 20 µm in tissue, respectively. Scans were obtained from 4.5-mm × 4.5-mm cubes, with each cube consisting of 320 clusters of 4 repeated B-scans centered on the optic disc. En-face projections of volumetric scans allowed the visualization of structural and vascular details within segmented layers of interest.

The microvasculature was evaluated in en-face images of the parapapillary deep layer generated based on the automated layer segmentation performed by the software controlling the operation of the OCT instrument. En-face images of the deep layer were derived from an en-face slab that extended from the RPE to 390 µm below BM, which was sufficient to include the full thickness of the choroid and the inner scleral surface.

A nonjuxtapapillary MvD was defined as a focal sectoral capillary dropout with decreased microvascular network present within the nonjuxtapapillary PPA area and distant from the optic disc margin identified in the deep-layer en-face images. Two independent observers (E.J.L. and G.N.K.) identified nonjuxtapapillary MvDs while blinded to the clinical information of the participants. Disagreements between the observers were resolved by a third adjudicator (T.W.K.). All of the included OCT B-scan images had image quality scores of ≥30, in accordance with the manufacturer's recommendation. An eye was excluded from the analysis when the quality of the OCTA images was poor (e.g., due to blurring) or when the vascular signal was blocked by artifacts (e.g., due to blinking or masking^15^).

### Evaluation of Parapapillary Microstructure

The optic disc including the parapapillary area was scanned by the Spectralis OCT system. The imaging scan covered a rectangular area of the optic disc (10° x 15°) that included approximately 65 sections separated by 30 to 34 mm. In total, 42 OCT frames were averaged for each section. The value of corneal curvature was entered into the Spectralis OCT system before the scan in order to remove the magnification error. Only eyes for which good-quality images (i.e., quality score ≥15) were obtained for all of the scans were included in the analysis.

The maximum radial width of the γ-zone was measured manually using a built-in caliper tool of the Spectralis OCT system (Heidelberg Eye Explorer software version 1.10.2.0; Heidelberg Engineering). A line was drawn connecting the disc center and BM opening at the furthest part from the optic disc center, and then the distance from the disc margin to the BM opening was measured on the infrared images in millimeters. The optic disc center was determined as the point where the long and short axes of the disc crossed. All measurements were made by two independent observers (E.J.L. and G.N.K.) who were blinded to the clinical information of the subjects. The average of two measurements made by each observer was used for the analyses.

### Data Analysis

The interobserver agreement confirming the presence of a nonjuxtapapillary MvD was assessed using kappa statistics (κ value). The presence of normality was tested using the Kolmogorov-Smirnov test. Demographic, ocular, and systemic characteristics were compared between groups using the independent-samples *t*-test and chi-square test for continuous and categorical variables, respectively. All of the statistical analyses were performed using SPSS software (version 19.0; SPSS, Chicago, IL, USA). The criterion for statistical significance was *P* < 0.05.

## Results

This study initially included 159 nonglaucomatous healthy myopic eyes with a γ-zone that underwent OCTA, from which 33 eyes were excluded due to poor OCTA image quality. An MvD was identified in 25 of the remaining 126 eyes (19.8%) in the distal portion of the PPA γ-zone. There was excellent interobserver agreement regarding the detection of a nonjuxtapapillary MvD (κ = 0.958).

### Parapapillary Microstructure According to the Presence or Absence of Nonjuxtapapillary MvD

**Figure 1.**
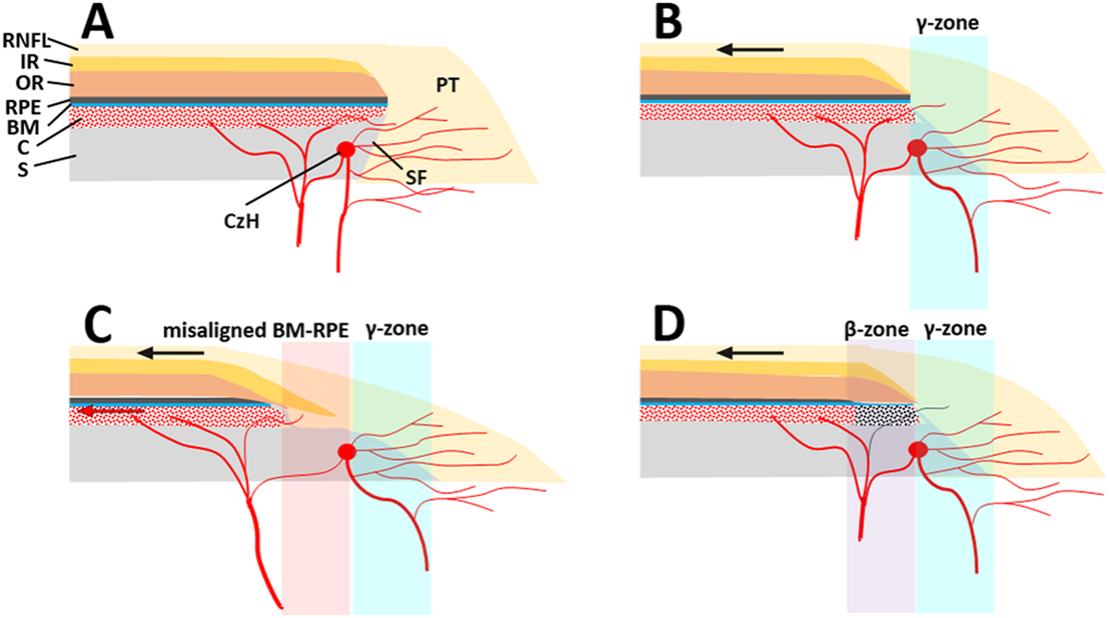
Schematic illustrations of various parapapillary structures. (**A**) Nonmyopic eye without a γ-zone. (**B**) Myopic eye with a γ-zone but no nonjuxtapapillary MvD. Myopic axial elongation stretches the temporal parapapillary tissues (*black arrow*), resulting in the oblique SF presenting as a clinical γ-zone (*light-blue region*). Note that the SF within the γ-zone is abundant in microvessels supplying the prelaminar tissue (PT). (**C**) Myopic eye with a γ-zone and nonjuxtapapillary MvD associated with a misaligned BM–RPE complex. The BM-RPE complex is misaligned temporally with the adjacent inner retina (IR) and choroid (C), and further stretching potentially exceeds the elastic limit of the BM-RPE complex (*red arrow*). Note that the parapapillary endpoints of the BM-PRE complex, outer retina (OR), and C do not merge with the other tissues, whereas the end points of all retinal layers and C merge at the end point of the *BM-RPE* complex in other cases (**A**, **B**, **D**). The separation between the Circle of Zinn-Haller (CzH) and the end point of the BM-RPE complex is also notable. Although microvessels are still abundant in the deep layer (i.e., SF) of the γ-zone (*light-blue region*), they are scarce in the deep layer (i.e., nonjuxtapapillary sclera [S]) at the location of the misaligned BM-RPE (*light-red region*). (**D**) Myopic eye with a γ-zone and nonjuxtapapillary MvD associated with a β-zone. Although the microstructure is similar to that in the eye without nonjuxtapapillary MvD (**B**), choroidal perfusion is decreased in the β-zone (*light-violet region*) presenting as nonjuxtapapillary MvD. Note that microvessels are still abundant within the γ-zone (*light-blue region*). RNFL = retinal nerve fiber layer; IR = inner retina; OR = outer retina; RPE = retinal pigment epithelium; BM = Bruch's membrane; C = choroid; S = sclera; CzH = Circle of Zinn-Haller; SF = scleral flange; PT = prelaminar tissue.

**Figure 2.**
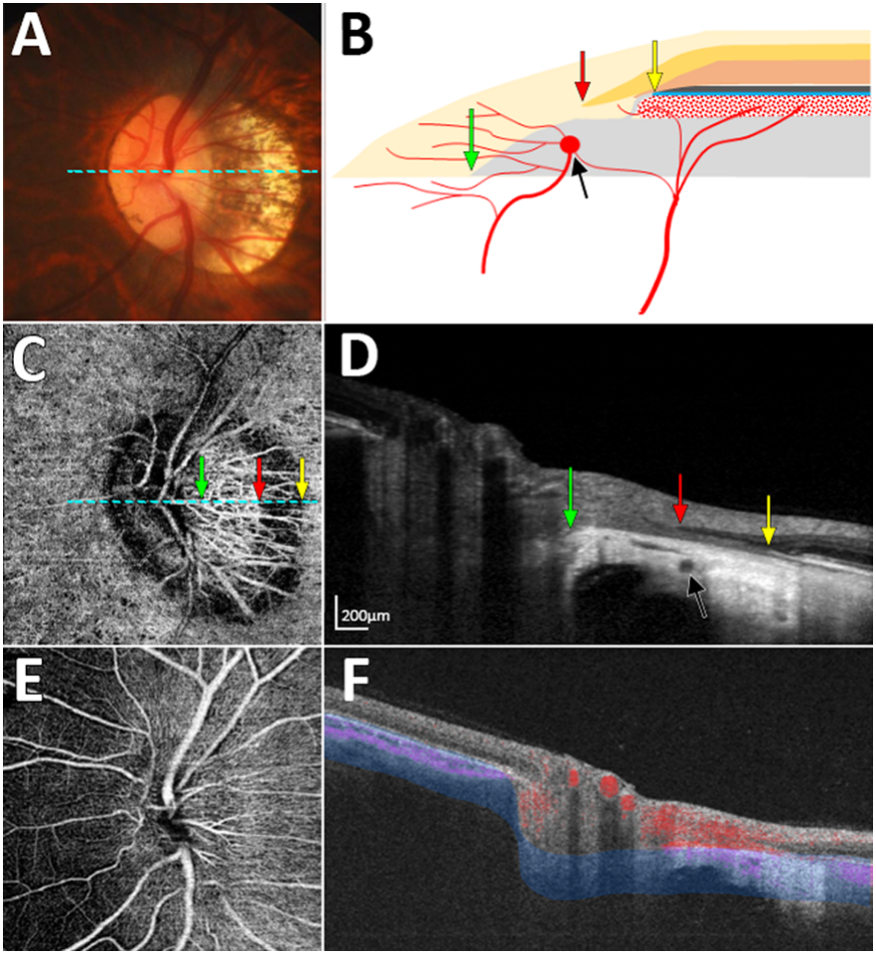
Case with nonjuxtapapillary microvasculature dropout (MvD) associated with misalignment of the Bruch's membrane (BM)–retinal pigment epithelium (RPE) complex. (**A**) Color disc photograph. (**B**) Schematic illustration showing misalignment of the BM-RPE complex. (**C**) En-face optical coherence tomography angiography (OCTA) image obtained in the parapapillary deep layer (*blue-area* in panel **F**), showing a nonjuxtapapillary MvD. (**D**) Horizontal B-scan image obtained at the center of the optic nerve (dashed lines in **A** and **C**). (**E**) En-face OCTA image obtained from superficial layer above the RPE. (**F**) OCTA B-scan Image showing the segmented layer (*blue-area*) to produce an en-face image of the deep layer in panel **C**. *Green*, *red*, and *yellow arrows* in panels **B**–**D** indicate the clinical optic disc margin and the inner and outer margins of the nonjuxtapapillary MvD, respectively. *Black arrows* indicate the circle of Zinn-Haller (CzH). Note that the nonjuxtapapillary MvD is located between the parapapillary endpoint of the inner retinal layer (**D**, *red arrow*) and the parapapillary end point of the BM-RPE complex (**D**, *yellow arrow*), and the CzH is located at the proximal end of the nonjuxtapapillary MvD (**D**, *black arrows*).

The parapapillary microstructure was evaluated in horizontal B-scan images. Figure 1 presents schematic illustrations of various parapapillary structures in the PPA γ-zone. The absence of the BM-RPE complex was a universal feature of the PPA γ-zone, but it could be further divided into two characteristic types based on the presence of inner retinal tissues in 18 of the 25 eyes with a nonjuxtapapillary MvD: (1) a more-proximal region consisting of prelaminar tissue and oblique SF (Fig. 1C), and (2) a more-distal region consisting of inner retinal layers and sclera (Fig. 1C). Although the former type was universally present in all eyes with a γ-zone (i.e., conventional γ-zone), the latter type was found only in the eyes with a nonjuxtapapillary MvD (*n* = 18), and was present at the location of the nonjuxtapapillary MvD. Only the BM-RPE complex and adjacent layers (choroid and outer retina) were not present in the latter structure, which indicated that the BM-RPE complex might have been dragged in a temporal direction along with adjacent tissues (i.e., misalignment of the BM-RPE complex). Such misalignment of the BM-RPE complex was defined as being present when the horizontal width of the structure exceeded 200 µm in horizontal B-scan images. This was based on our previous study defining the γ-zone as the horizontal width of externally oblique border tissue exceeding 200 µm.^6^ In eyes with a nonjuxtapapillary MvD but without such misalignment of the BM-RPE complex (*n* = 7), the location of the nonjuxtapapillary MvD corresponded to where RPE atrophy was present (i.e., conventional β-zone; Fig. 1D). Hypointense signals indicating the Circle of Zinn-Haller were observed in 22 eyes at the proximal margin of the nonjuxtapapillary MvD (Figs. 1C, 1D).

**Figure 3.**
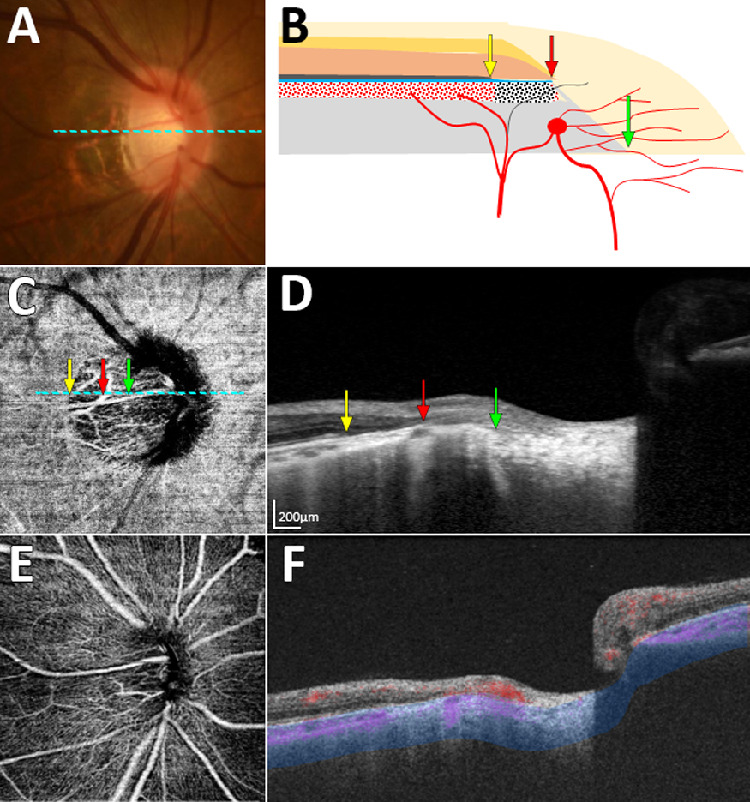
Case with nonjuxtapapillary microvasculature dropout (MvD) not associated with misalignment of the Bruch's membrane (BM)–retinal pigment epithelium (RPE) complex. (**A**) Color disc photograph. (**B**) Schematic illustration showing an atrophic RPE (β-zone) without misalignment of the BM-RPE complex at the location of the nonjuxtapapillary MvD. (**C**) En-face optical coherence tomography angiography (OCTA) image obtained in the parapapillary deep layer (*blue-area* in panel **F**), showing a nonjuxtapapillary MvD. (**D**) Horizontal B-scan image obtained at the center of the optic nerve (dashed lines in **A** and **C**). (**E**) En-face OCTA image obtained from superficial layer above the RPE. (**F**) OCTA B-scan Image showing the segmented layer (*blue-area*) to produce an en-face image of the deep layer in panel **C**. *Green*, *red*, and *yellow arrows* in panels **B**–**D** indicate the clinical optic disc margin and the inner and outer margins of the nonjuxtapapillary MvD, respectively. Note that all retinal layers and the choroidal layer merge at the termination of the BM-RPE complex (**D**, *red arrow*). The location of the nonjuxtapapillary MvD corresponds to that of the β-zone area, which spans within the terminations of the RPE (**D**, *yellow arrow*) and BM (**D**, *red arrow*).

**Figure 4.**
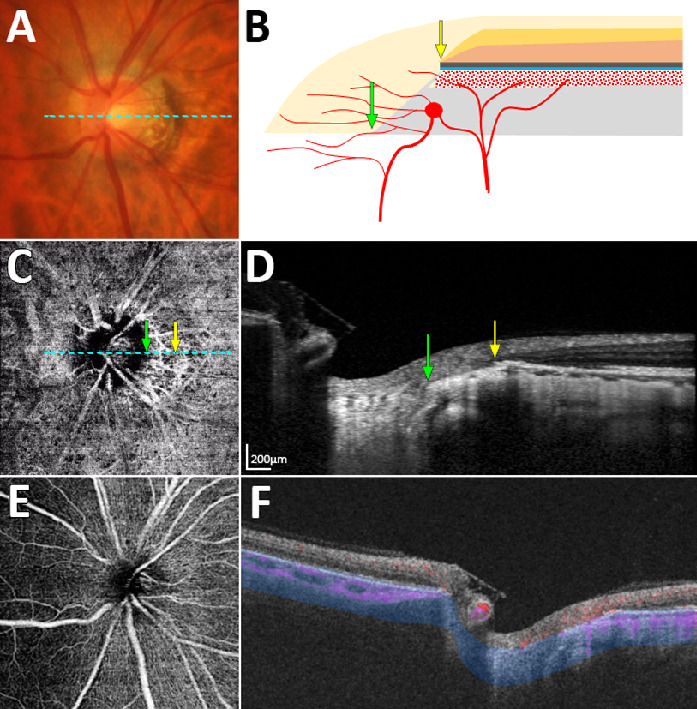
Case without microvasculature dropout. (**A**) Color disc photograph. (**B**) Schematic illustration showing conventional γ-zone without misalignment of the Bruch's membrane (BM)–retinal pigment epithelium (RPE) complex. (**C**) En-face optical coherence tomography angiography (OCTA) image obtained in the parapapillary deep layer (*blue-area* in panel **F**). (**D**) Horizontal B-scan image obtained at the center of the optic nerve (dashed lines in **A** and **C**). (**E**) En-face OCTA image obtained from superficial layer above the RPE. (**F**) OCTA B-scan Image showing the segmented layer (*blue-area*) to produce an en-face image of the deep layer in panel **C**. *Green* and *yellow arrows* in panels **B**–**D** indicate the clinical optic disc margin and the outer margin of the parapapillary atrophy γ-zone, respectively. Neither misalignment of the BM-RPE complex nor atrophy of RPE are observed near the γ-zone (**D**).

**Figure 5.**
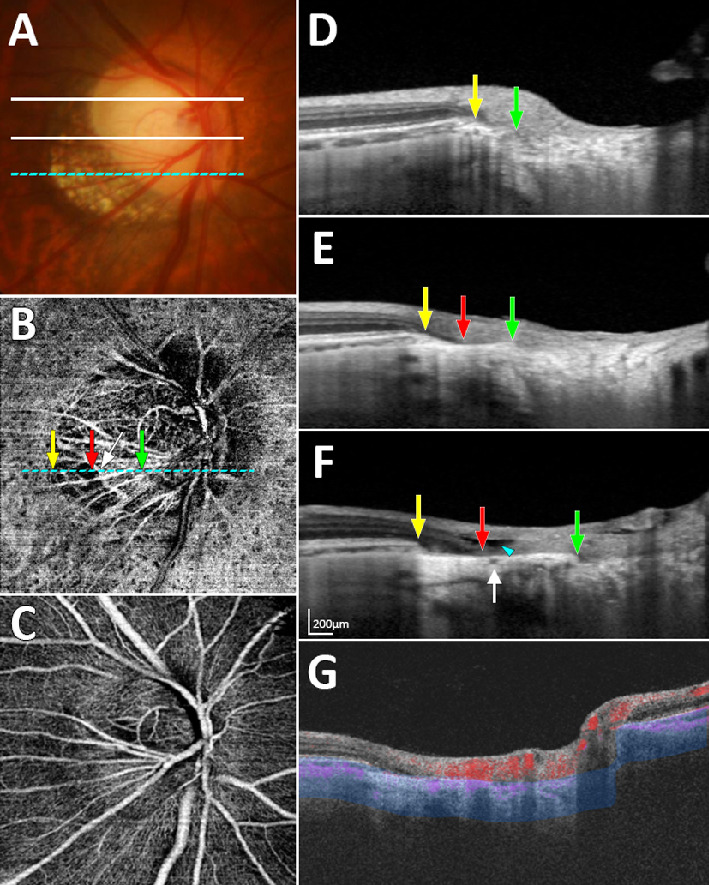
Case shown in the Supplementary Video, which exhibited a nonjuxtapapillary microvasculature dropout (MvD) and misalignment of the Bruch's membrane (BM)–retinal pigment epithelium (RPE) complex. (**A**) Color disc photograph indicating the three locations where the horizontal B-scans in panels **D**, **E**, and **F** were obtained. (**B**) En-face optical coherence tomography angiography (OCTA) image obtained in the parapapillary deep layer (*blue-area* in panel **G**), showing a nonjuxtapapillary MvD in the inferotemporal parapapillary area. (**C**) En-face OCTA image obtained from superficial layer above the RPE. (**D**–**F**) Horizontal B-scan images obtained at the locations indicated in panel **A**. (**G**) OCTA B-scan Image showing the segmented layer (*blue-area*) to produce an en-face image of the deep layer in panel **B**. *Green*, *red*, and *yellow arrows* in panels **B**, **D**, **E**, and **F** indicate the clinical optic disc margin and the inner and outer margins of the nonjuxtapapillary MvD, respectively. *White arrow* indicates the circle of Zinn-Haller. Note that the nonjuxtapapillary MvD is located between the parapapillary endpoint of inner retinal layer (**F**, *red arrow*) and the parapapillary end point of the BM-RPE complex (**F**, *yellow arrow*). The hyporeflective space between the inner and outer retina (**F**, *arrowhead*) suggests different responses of the inner and outer retina to temporal stretching. More superiorly to the area of the MvD, the oblique scleral flange (**D**, area between *green* and *yellow arrows*) is identified (conventional γ-zone) but no misalignment of the BM-RPE complex is evident.

Figures 2 and 3 show representative cases of eyes having a nonjuxtapapillary MvD: one with and one without a misaligned BM-RPE. Figure 4 shows a representative case of an eye without a nonjuxtapapillary MvD. The regional differences in the parapapillary microstructure according to the presence or absence of the nonjuxtapapillary MvD can be clearly identified in the Supplementary Video. Figure 5 is the same eye shown in that video.

### Comparison of Clinical Characteristics Between Eyes with and without Nonjuxtapapillary MvD

Eyes with a nonjuxtapapillary MvD (*n* = 25) were compared with those without any MvD (*n* = 25) age-matched (within 5 years) on a 1:1 basis. Table 1 compares the clinical characteristics: eyes with a nonjuxtapapillary MvD had a longer AXL (26.8 [1.5] vs. 25.8 [1.4] mm; *P* = 0.013) and a larger maximum γ-zone width (754.6 [301.8] vs. 335.2 [130.5] µm; *P* < 0.001) than those without any MvD, and a misaligned BM-RPE was observed only in the eyes with a nonjuxtapapillary MvD.

**Table 1.** Comparison of Clinical Characteristics Between the Healthy Myopic Eyes With and Without Nonjuxtapapillary MvD

	Eyes With Nonjuxtapapillary MvD (*N* = 25)	Eyes Without Nonjuxtapapillary MvD (*N* = 25)	*P* Value
Age, y	44.2 ± 12.7	43.1 ± 11.8	0.740^*^
Sex, male/female	12/13	12/13	1.000^†^
IOP at OCTA exam, mm Hg	13.0 ± 3.1	13.8 ± 2.4	0.293^*^
Spherical error, diopters	−6.7 ± 3.4	−4.6 ± 3.4	**0.037** ^*^
Axial length, mm	26.8 ± 1.5	25.8 ± 1.4	**0.013** ^*^
Maximum γ-zone width, µm	754.6 ± 301.8	335.2 ± 130.5	**<** 0.001^*^
Central corneal thickness, µm	543.4 ± 49.6	567.4 ± 42.6	0.072^*^
Global RNFL thickness, µm	89.3 ± 7.4	92.0 ± 6.7	0.193^*^
Misalignment of BM-RPE, no. (%)	18 (72.0)	0 (0.0)	**<** **0.001**^†^
Presence of β-zone, no. (%)	9 (36.0)	7 (28.0)	0.544^†^

MvD, microvasculature dropout; IOP, intraocular pressure; OCTA, OCT angiography; RNFL, retinal nerve fiber layer; BM, Bruch's membrane; RPE, retinal pigment epithelium.

Boldface indicates statistical significance.

* Statistical comparison was performed using independent samples *t*-test.

† Statistical comparison was performed using chi-square test.

### Comparison of Clinical Characteristics of the Eyes with Nonjuxtapapillary MvD According to the Presence or Absence of a Misaligned BM-RPE

The eyes with a misaligned BM-RPE were younger (39.8 [9.4] vs. 55.6 [13.7] years; *P* = 0.006), had a lower IOP (12.1 [2.8] vs. 15.1 [3.0] mm Hg; *P* = 0.025), and had a larger maximum width of the γ-zone (839.1 [300.9] vs. 537.4 [176.7] mm; *P* = 0.014) than those without a misaligned BM-RPE (Table 2). A PPA β-zone occurred more frequently in the eyes without a misaligned BM-RPE (100.0% vs. 11.1%; *P* < 0.001; Table 2).

**Table 2.** Comparison of Clinical Characteristics According to the Presence and Absence of Misaligned BM-RPE in the Healthy Myopic Eyes With Nonjuxtapapillary MvD

	Eyes With Misaligned BM-RPE (*N* = 18)	Eyes Without Misaligned BM-RPE (*N* = 7)	*P* Value
Age, y	39.8 ± 9.4	55.6 ± 13.7	**0.006** ^*^
Sex, male/female	9 / 9	3 / 4	0.748^†^
IOP at OCTA exam, mm Hg	12.1 ± 2.8	15.1 ± 3.0	**0.025** ^*^
Spherical error, diopters	−6.9 ± 3.8	−5.9 ± 2.3	0.534^*^
Axial length, mm	27.1 ± 1.6	26.3 ± 1.0	0.270^*^
Maximum γ-zone width, µm	839.1 ± 300.9	537.4 ± 176.7	**0.014** ^*^
Central corneal thickness, µm	544.6 ± 37.6	540.3 ± 76.3	0.326^*^
Global RNFL thickness, µm	89.36 ± 6.0	88.7 ± 10.9	0.534^*^
Presence of β-zone, no. (%)	2 (11.1)	7 (100)	**<** **0.001**^†^

MvD, microvasculature dropout; BM, Bruch's membrane; RPE, retinal pigment epithelium; IOP, intraocular pressure; OCTA, OCT angiography; RNFL, retinal nerve fiber layer.

* Statistical comparison was performed using independent Mann-Whitney test.

† Statistical comparison was performed using chi-square test.

## Discussion

This study has revealed nonjuxtapapillary MvDs in the parapapillary deep layer located in the distal portion of the PPA γ-zone in healthy myopic eyes. A nonjuxtapapillary MvD was observed in 25 of the 126 analyzed healthy myopic eyes. The parapapillary microstructure at the nonjuxtapapillary MvD was characterized in approximately 70% of eyes by the absence of the BM-RPE complex, choriocapillaris, and outer retinal layers, and the presence of inner retinal layers and sclera, which were distinctive features that were not observed in the eyes without any MvDs.

The structural differences between the nonjuxtapapillary MvD and the MvD in the γ-zone in glaucomatous eyes^9^^,^^10^ suggest that different pathogeneses underlie these two types of MvD. The MvD reported in glaucoma adjoins the optic disc margin and it is considered a dropout of microvessels within the SF, which may supply the prelaminar ONH tissue. Studies have shown that such MvD in glaucoma is associated with a longer AXL and larger PPA γ-zone,^9^^,^^10^ suggesting that it is associated with the tensile stress imposed on the SF and the microvasculature within it.

However, the nonjuxtapapillary MvD in our study was observed at the more-distal sclera rather than at the SF, and was associated with misalignment of the BM-RPE complex. The parapapillary end point of the BM-RPE complex is usually connected to the end of the parapapillary choroidal border tissue of Elschnig, which separates the choroidal space from the intrapapillary space of the ONH,^16^ and the end point of the retinal layer merges at the end point of the BM-RPE complex. In 18 of the 25 eyes with a nonjuxtapapillary MvD, the end point of the BM-RPE complex was displaced temporally and did not meet the end point of the retinal layer. The Circle of Zinn-Haller in eyes without a high degree of myopia is located at the point where the dura mater merges with the sclera at the end of the peripapillary SF.^17^ The location of the Circle of Zinn-Haller at the proximal end of the nonjuxtapapillary MvD suggested that the BM-RPE complex was displaced also from the point where it merges with the SF, resulting in sclera exposure (Fig. 1C). This structure differed from a conventional γ-zone, in which the location where the retina, BM-RPE complex, and the border tissue merge is preserved (Fig. 1B). More proximal to the nonjuxtapapillary MvD, the parapapillary area consisted of prelaminar tissue and the SF regardless of the presence of a misaligned BM-RPE (Figs. 1C, 1D), which is similar to the structure of the γ-zone in the eyes without any MvD (Fig. 1B).

The γ-zone is considered a product of an exposed SF resulting from stretching of the temporal peripapillary tissues in the course of myopic axial elongation (Figs. 1A, 1B).^8^^,^^18^^–^^20^ However, the peripapillary tissues comprise several layers with different elasticities^21^^,^^22^ (e.g., retina, BM, choroid, and sclera) that extend to the ONH tissues in different ways. When the stretching tension exceeds the combined elastic limit of the tissues, it is likely that the less-elastic tissue with a weaker connection to the ONH will be the first to become disrupted or dislocated. We speculate that the BM-RPE complex and the layers attached to it are prone to this disruption and will be dragged temporally from the other peripapillary tissues when subjected to excessive stretching (Fig. 1C). On the other hand, the sclera is more elastic and extends toward the SF, which attaches to the lamina cribrosa, and the inner retina is an extension of the prelaminar tissue. Hence, the sclera and inner retina might tend to remain in place despite the presence of excessive stretching. This hypothesis is supported by a longer AXL and larger maximum γ-zone width being associated with the presence of a nonjuxtapapillary MvD, and also by recent longitudinal studies demonstrating the papillary and parapapillary structural changes associated with myopia progression.^23^^–^^25^ However, dragging of the BM-RPE complex could not be confirmed by this cross-sectional study. Therefore, the characteristic structure associated with the nonjuxtapapillary MvD was described as “misalignment” in the present study, rather than as “dragging” of the BM-RPE complex.

A focally detached inner retina is evident at the location of the nonjuxtapapillary MvD in the eye shown in Figure 5E. We speculate that this indicates different responses of the various layers to temporal stretching, and the inner retina not being extended as much as the sclera. The stress induced by this extension might result in damage to the retinal ganglion cell axons, and may explain why myopic eyes are susceptible to glaucomatous damage. A future longitudinal study might be able to determine whether these eyes are more prone to glaucoma.

The concept of misalignment of the BM-RPE complex is consistent with the histological study of Jonas et al.^26^ Those authors observed a corrugated BM at its peripapillary termination in highly myopic eyes with a large γ-zone, and proposed that the BM corrugation represents the rupture or disinsertion of BM from the border tissue as a consequence of an increased tension within the BM. In this context, the presence of a nonjuxtapapillary MvD might indicate the past or current presence of tensile stress. Whether such tensile stress associated with the nonjuxtapapillary MvD could influence certain pathological processes remains to be determined in longitudinal studies.

Jonas et al.^27^ demonstrated a defective BM in the macular area, which was strongly associated with a longer AXL in highly myopic eyes. Those authors suggested that marked stretching of the globe in high myopia can lead to a defective BM in the macula. None of our subjects showed macular BM defects, which might have been due to our exclusion of eyes with extreme myopia having retinal pathology. The AXL was indeed shorter in our study (26.8 ± 1.5 mm, mean ± SD) than that reported by Jonas et al.^27^ (30.0 ± 2.8 mm).

We do not have a clear answer for how this distinctive structure presented as a decreased microvasculature in the OCTA image of the parapapillary deep layer. It is possible that the nonjuxtapapillary MvD was identifiable due to the microvascular density differing between the juxtapapillary SF and the nonjuxtapapillary sclera (Fig. 1C). The juxtapapillary SF has a relatively abundant microvasculature originating from the short posterior ciliary artery, which supplies the lamina cribrosa and prelaminar tissues via the circle of Zinn-Haller. However, the microvasculature is less densely distributed in the nonjuxtapapillary sclera. Therefore, OCTA could have distinguished the area of nonjuxtapapillary sclera from that of the oblique SF based on the difference in the microvascular density in the eyes with a misaligned BM-RPE.

Seven of the 25 eyes with a nonjuxtapapillary MvD did not show a dragged BM-RPE, but the nonjuxtapapillary MvD was characterized by RPE atrophy (i.e., PPA β-zone^6^^,^^7^), which has been suggested as an area with decreased choroidal blood flow.^28^^–^^31^ Sung et al.^31^ reported that the deep-layer vessel density in OCTA was lower in the PPA β-zone than in the PPA γ-zone in young myopic eyes. It can be speculated that decreased choroidal perfusion in the β-zone appeared as a nonjuxtapapillary MvD in these eyes. It has been shown that the PPA β-zone is significantly associated with faster glaucoma progression.^32^^–^^34^ Breakdown of the blood–optic-nerve barrier and a reduced blood supply in the β-zone area have been proposed as possible mechanisms underlying such a relationship.^35^^,^^36^ It is intriguing that the β-zone was also present in the eyes without any MvD (*n* = 7). It would be of interest to understand the clinical significance of the nonjuxtapapillary MvD associated with the β-zone.

Eyes with a nonjuxtapapillary MvD associated with the β-zone were older than those with a dragged BM-RPE, which is partially compatible with a previous study finding that the PPA β-zone was associated with older age in patients with primary open-angle glaucoma.^6^ Those eyes also had a higher IOP than those with a dragged BM-RPE. It is possible that age-related RPE atrophy and the IOP together affect the development of a β-zone and decreased vascularity within the β-zone. In this context, it can be speculated that the nonjuxtapapillary MvD associated with RPE atrophy probably results from increased pressure, while that associated with a dragged BM-RPE is more likely to result from stretching.

This study was subject to several limitations. First, because it had a cross-sectional design, we could not determine the pathogenic role of the nonjuxtapapillary MvD. A long-term prospective study might be needed to clarify the clinical significance of our findings. Second, detection of the peripapillary endpoint of the BM using OCT is often difficult in high myopia, when the interface between the BM and the choroidal layer becomes indistinct.^37^ In addition, it is not easy to distinguish BM from the RPE based on OCT. Therefore, our observation should be confirmed by further histologic studies. Third, projection artifacts caused by the moving shadows cast by blood cells^38^^,^^39^ flowing in the overlying retinal vessels hamper the precise definition of an MvD boundary. The software provided with the DRI-OCT Triton system unfortunately does not include an algorithm to remove such projection artifacts.

In conclusion, nonjuxtaparapapillary MvDs were found in the distal portion of the PPA γ-zone in nonglaucomatous healthy myopic eyes. The underlying parapapillary microstructure, which was characterized by the absence of the BM-RPE complex with overlying inner retina, suggested that the BM-RPE complex had been dragged temporally, possibly due to stretching. In the eyes without such a misaligned BM-RPE complex, the β-zone was present in the area with the nonjuxtapapillary MvD. The nonjuxtapapillary MvD should be differentiated from the choroidal MvD observed in glaucomatous eyes based on the characteristic features. Further studies are needed to determine the clinical significance of the nonjuxtapapillary MvDs with different microstructures.

## Supplementary Material

Supplementary Video En-face optical coherence tomography angiography image obtained at parapapillary deep layer (left), and serial horizontal B-scan images of the optic nerve head (right) demonstrate regional relationship between nonjuxtapapillary microvasculature dropout (MvD) and misalignment of the Bruch's membrane (BM)-retinal pigment epithelium (RPE) complex. *Green*, *red*, and *yellow arrows* indicate the clinical optic disc margin and the inner and outer margins of the nonjuxtapapillary MvD, respectively. Note that the nonjuxtapapillary MvD is located between the parapapillary endpoint of inner retinal layer (*red arrow*) and the parapapillary endpoint of the BM-RPE complex (*yellow arrow*).
